# The Geomagnetic Field (GMF) Is Necessary for Black Garden Ant (*Lasius niger* L.) Foraging and Modulates Orientation Potentially through Aminergic Regulation and *MagR* Expression

**DOI:** 10.3390/ijms24054387

**Published:** 2023-02-23

**Authors:** Giuseppe Mannino, Luca Pietro Casacci, Giorgia Bianco Dolino, Giuseppe Badolato, Massimo Emilio Maffei, Francesca Barbero

**Affiliations:** Department of Life Sciences and Systems Biology, University of Turin, Via Accademia Albertina 13/Via Gioacchino Quarello 15/A, 10123 Torino, Italy

**Keywords:** nearly null magnetic field, dopamine, serotonin, melatonin, phylogenetic analysis, antioxidant enzymes, oxidative stress, *Lasius niger*

## Abstract

The geomagnetic field (GMF) can affect a wide range of animal behaviors in various habitats, primarily providing orientation cues for homing or migratory events. Foraging patterns, such as those implemented by *Lasius niger*, are excellent models to delve into the effects of GMF on orientation abilities. In this work, we assessed the role of GMF by comparing the *L. niger* foraging and orientation performance, brain biogenic amine (BA) contents, and the expression of genes related to the magnetosensory complex and reactive oxygen species (ROS) of workers exposed to near-null magnetic fields (NNMF, ~40 nT) and GMF (~42 µT). NNMF affected workers’ orientation by increasing the time needed to find the food source and return to the nest. Moreover, under NNMF conditions, a general drop in BAs, but not melatonin, suggested that the lower foraging performance might be correlated to a decrease in locomotory and chemical perception abilities, potentially driven by dopaminergic and serotoninergic regulations, respectively. The variation in the regulation of genes related to the magnetosensory complex in NNMF shed light on the mechanism of ant GMF perception. Overall, our work provides evidence that the GMF, along with chemical and visual cues, is necessary for the *L. niger* orientation process.

## 1. Introduction

The geomagnetic field (GMF) is one of the abiotic components that has interacted continuously with living organisms since the beginning of life on Earth [1]. All living organisms are affected by the GMF, from bacteria to plants [2,3,4], up to invertebrates [5,6] and vertebrates [7,8,9,10]. Birds can use the GMF vectors during homing or migratory events as a compass for orientation [11]. Four different mechanisms of magnetoperception have been described: (i) the radical pair mechanism (i.e., magnetically sensitive chemical intermediates that are formed by photoexcitation of cryptochrome [12,13], which is present in animals [14], humans [15], and plants [16]); (ii) the presence of magnetic field (MF) sensory receptors described in magnetotactic bacteria [17]; (iii) the presence of electroreceptors in elasmobranch animals [18]; (iv) the biocompass model based on the MagR/Cry complex, demonstrated in the model insect *Drosophila melanogaster* [19]. Among the four possible mechanisms of magnetoreception, at least two (the radical pair mechanism of chemical magnetosensing and the MagR/Cry biocompass) adequately explain the alterations in the MF by the rates of redox reactions and subsequently altered concentrations of free radicals and reactive oxygen species (ROS) observed in different organisms [16,20,21,22].

Ants and other social insects could represent outstanding models to study the effects of GMF on orientation patterns. Because they live in colonies, sometimes counting hundreds of individuals, their persistence and success rely on a complex organization maintained through a multimodal communication system [23,24]. The needs of the whole colony are fulfilled by collective decision making, coordinating, and regulating several workers engaged in distinct labors without central control [23]. Thus, ants show a wide variety of foraging strategies to optimize the resource intake for the whole colony. The efficiency of the foraging behavior is strictly linked to the ant’s orientation abilities which can be based on “egocentric” or “geocentric” cues [25,26], or by a combination of the two reference systems during homing or food searching. The former includes proprioceptive signals continuously obtained while walking out and integrated to return to the nest, while the latter is landmark-based information (visual or chemical) used to infer the target position [26,27].

*Lasius niger* L. (black garden ant) workers show a plastic foraging strategy based on the combination of several pieces of information, such as pheromone trails, visual cues, and encounters with nestmates [28,29,30,31,32]. Although the foraging behavior of black garden ants has been extensively studied (see [32] and references therein), to our knowledge, there is no experimental evidence of a potential role of the GMF on their orientation. 

To evaluate the effect of GMF on the orientation abilities of *L. niger*, we used a triaxial Helmholtz coils system able to reduce the local GMF (42.20 ± 0.02 µT) to near-null magnetic field (NNMF, 45 ± 6 nT) values and observed how ant foraging performance varied. The system used (see [33] for a technical explanation) allows rearing ants at the same light, temperature, humidity, and magnetic field (MF) inclination and declination as the GMF controls, varying only the MF intensity. Because biogenic amines (BAs), which act as neurotransmitters, neuromodulators, or neurohormones, are involved in the ant behavioral plasticity [34,35,36], we tested the GMF-dependent aminergic regulation of ant foraging behavior by reducing the GMF to NNMF conditions. Recently, the study of BAs has attracted great interest in entomology, as they are responsible for behavioral modifications, including muscle performance, locomotory, learning processes, memory, aggression, and both aggressive and nonaggressive social interactions [37,38]. Among other BAs, tyramine (TA), dopamine (DA), l-3,4-dihydroxyphenylalanine (L-DOPA), and serotonin (Ser) are well known as essential transmitters not only in vertebrates but also in invertebrates [35,37,39]. DA and L-DOPA are involved in food-searching behavior, while Ser antagonizes the effects of octopamine (OA) in the control of rhythmic behaviors, but it is more entailed in learning and memory processes [35,37,40,41]. In invertebrates, melatonin (Mel) has been recently investigated in abiotic tolerance to stress [39,42], whereas TA and OA, which have no physiological significance in vertebrates, play essential roles in controlling different behaviors, including muscle contraction and sense organ sensitivity [34].

Here, we show that reducing GMF to NNMF decreases workers’ orientation performances, potentially through dopaminergic and serotoninergic regulations. Moreover, we show that the regulation of genes related to the magnetosensory complex [43,44] implies the perception of GMF in black garden ants. Overall, we provide evidence that the GMF, along with chemical and visual cues, is pivotal in the *L. niger* orientation process.

## 2. Results and Discussion

### 2.1. The GMF Is Necessary for the Orientation Abilities of Lasius niger

During foraging, *L. niger* gathers spatial information by combining chemical and visual cues [45]; therefore, we designed our bioassays to deliberately include both chemical trails and conspicuous landmarks, along with the reduction in the GMF. Increasing or reverting the MF has been correlated with significant changes in ants’ foraging strategies, leading to variations in the path trajectories, as revealed for *Pheidole* sp. [46], or increasing the time necessary to lay trails in *Solenopsis invicta* [47], suggesting the ants’ ability to respond to MF variations by perceiving or releasing chemical signals [46]. However, the use of Helmholtz coils placed directly in the arenas or mazes used for the bioassays might generate short-range environmental variations such as temperature increase [46,48], thus creating confounding factors. Our experimental approach does not introduce other variations, such as temperature increase, compared to the GMF conditions within the artificial arenas, but the reduction to NNMF values. 

**Figure 1:**
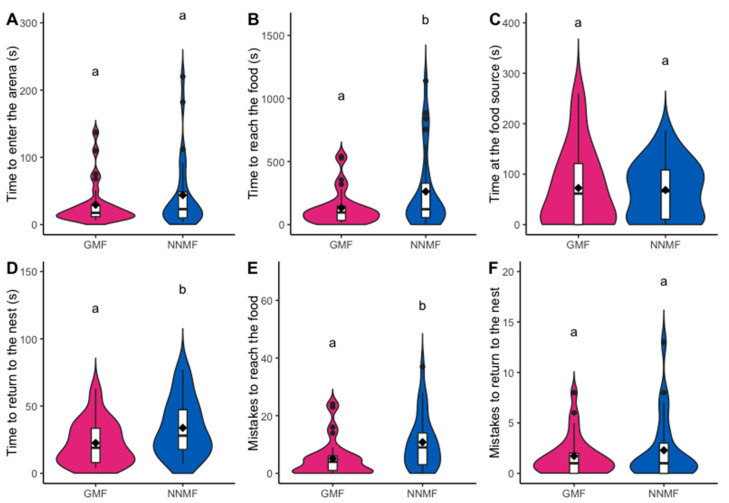
Foraging activity of *Lasius niger* under GMF and NNMF recorded during Phase 1. Violin plots show (**A**) the time spent by the first forager to enter the arena, (**B**) the time spent to reach the food source, (**C**) the time spent at the food source, (**D**) the time spent to return to the nest, (**E**) the number of mistakes made to reach the food source, and (**F**) the number of mistakes to return to the nest during Phase 1. The violin shape shows the distribution of the values, and the box plot inside is made by a black line in the center that indicates the median; the top and bottom of the black box represent the upper and lower quartiles; whiskers represent the maximum and minimum values, while the black dots indicate the outliers. Diamonds indicate mean values. Different letters indicate significantly (*p* < 0.05) different values based on generalized linear mixed model results.

In NNMF (Phase 1), *L. niger* workers took on average 43.78 ± 10.37 s to enter the arena by leaving their colony, while, in GMF, the time was 29.50 ± 6.00 s; however, this difference was not statistically significant (GLMM, LR χ^2^_1,55_ = 0.310, *p* = 0.578) (Figure 1A). Furthermore, we did not record any significant difference in the time spent at the food source by the first worker who started the foraging activity between NNMF and GMF conditions (GLMM, LR χ^2^_1,54_ = 0.0168, *p* = 0.897) (Figure 1C). The latter observation is in contrast with the findings of Pereira and coworkers [46], who found differences when applying a static MF of 60 µT. Therefore, the experimental conditions we used in our system neither stressed worker ants nor generated any disturbance in the ant ability to forage. However, we found that GMF plays a crucial role in *L. niger* orientation. Even if all other cues allowing the ant orientation were present, in NNMF, workers took twice as much time (262.04 ± 60.02 s; Video S1) to discover the food source (LMM, LR χ^2^_1,56_ = 6.251, *p* = 0.012) compared to workers in GMF (132.10 ± 25.54 s) (Figure 1B; Video S2), and the first worker took on average longer time (62.75 ± 20.94 s) to return from the food source to the center of the arena, with respect to the colonies observed in GMF (22.56 ± 3.12 s) (LMM, LR χ^2^_1,51_ = 5.385, *p* = 0.020) (Figure 1D). By considering the average number of errors made along the way (i.e., the number of times that the forager walked in a labyrinth arm that did not lead to the food), we found that, in NNMF, the first worker made more errors to reach the food source (10.82 ± 1.81) than in GMF (5.14 ± 1.21) (GLMM, LR χ^2^_1,56_ = 5.917, *p* = 0.015) (Figure 1E). In addition, the number of workers who made mistakes was extremely variable in NNMF and lower in GMF (Figure 1E). On the other hand, no significant differences (GLMM, LR χ^2^_1,51_ = 0.0150, *p* = 0.903) were observed between GMF and NNMF in the number of errors made by the first forager while returning to the nest after feeding at the food source, even if the returning time was longer (Figure 1F). It is, therefore, possible that NNMF conditions affected the locomotory ability of the first scouting worker, by extending the return walk time. However, the correct path could still be found on the basis of visual cues, because *L. niger* ants make a reduced use of chemical trails in the first phase of foraging [45,49].

**Figure 2:**
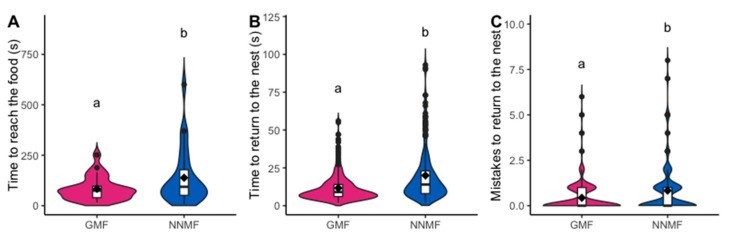
Foraging activity of *Lasius niger* under GMF and NNMF recorded during Phase 2. Violin plots show (**A**) the time spent by the first recruited worker to reach the food source, (**B**) the time to return to the nest by all workers that had fed for more than 30 seconds at the food source, and (**C**) the number of errors made while returning to the nest. The violin shape shows the distribution of the values, and the box plot is made by a black line in the center that indicates the median; the top and bottom of the black box represent the upper and lower quartiles; whiskers represent the maximum and minimum values, while the black dots indicate the outliers. Diamonds indicate mean values. Different letters indicate significantly (*p* < 0.05) different values based on generalized linear mixed model results.

After the first worker returned to the colonies, all nest members were allowed to enter the experimental arena leading to a collective foraging, according to the exchange of signals between nestmates and the deposition and perception of pheromone trails (so-called Phase 2) [49]. During Phase 2 of the experiment, the first recruited worker took, on average, significantly longer to reach the food source in NNMF (Video S3) than those in colonies exposed to GMF (GLMM, LR χ^2^_1,52_ = 5.651, *p* = 0.017) (Figure 2A; Video S4). By following all workers who fed at the food source for at least 30 s, we observed that foragers took an average longer time to return to the center of the arena in NNMF, compared to workers in GMF (LMM, LR χ^2^_1,498_ = 20.095, *p* < 0.001) (Figure 2B). Even the number of errors made by the workers while returning to the nest was found to be significantly higher in NNMF (GLMM, LR χ^2^_1,498_ = 14.777, *p* < 0.001) (Figure 2C). 

The best models (ΔAICc < 4) explaining the time to return to the nest during Phase 2 included the presence/absence of the GMF, the number of errors made by the foragers while returning to the nest, the interaction between these two terms, and the worker sequential order of arrival at the center of the arena (see Supplementary Table S1). The first model contained all entered terms, and only the presence and absence of the GMF and the errors made were statistically significant (GLMM, GMF/NNMF: LR χ^2^_1,498_ = 10.555, *p* = 0.001; errors: LR χ^2^_1,498_ = 56.446, *p* = 5.775e^−14^; GMF/NNMF × errors: LR χ^2^_1,498_ = 2.138, *p* = 0.144; order of arrival: LR χ^2^_1,498_ = 3.221, *p* = 0.073). As expected, the return time to the center of the arena increased as more errors were made along the way; however, the MF presence is the most important explanatory variable, providing other supporting evidence that the GMF is a key factor for the orientation abilities of *L. niger*.

**Figure 3:**
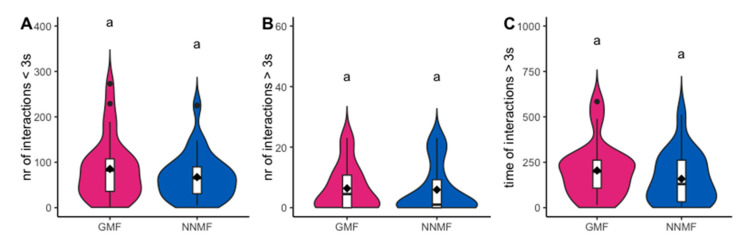
Interaction events of *Lasius niger* under GMF and NNMF recorded during Phase 2. Violin plots show (**A**) the number of interactions lasting less than 3 seconds between two workers, (**B**) the number of interactions lasting more than 3 seconds between two workers, and (**C**) the time of interactions lasting more than 3 s between two workers. The violin shape shows the distribution of the values, and the box plot inside is made by a black line in the center that indicates the median; the top and bottom of the black box represent the upper and lower quartiles; whiskers represent the maximum and minimum values, while the black dots indicate the outliers. Diamonds indicate mean values. Different letters indicate significantly (*p* < 0.05) different values based on generalized linear mixed model results.

During Phase 2, workers present in the arena interacted with each other. Many interactions (76.5 ± 8.3 events) occurred in GMF conditions between two workers and were found to be instantaneous, i.e., lasting less than 3 s. The workers generally interacted by touching each other with their antennae. In a limited number of cases (6.2 ± 1.1), two workers spent more time in the interaction, which involved, in addition to the antennating behavior, food exchange and allogrooming. The time of these prolonged interactions ranged from a few seconds to a few minutes. NNMF did not affect the number of interactions, both short (GLMM, LR χ^2^_1,53_ = 1.0624, p = 0.303) (Figure 3A) and prolonged (GLMM, LR χ^2^_1,52_ = 0.0296, df = 1, p = 0.863) (Figure 3B), or the time spent by the two workers in prolonged interactions (GLMM, LR χ^2^_1,36_ = 0.615, *p* = 0.433) (Figure 3C). These interaction events among workers were monitored because they can mediate the transfer of cues that signal the food position or quality [32,50,51]. Indeed, in *L. niger*, no evidence supports an exchange of information through this modality, while the contact between the antennae of two workers could serve to recognize the nestmates rather than transferring food information [31]. Our data provide indirect evidence to support this last hypothesis, as no significant differences were found in any types of exchange analyzed in either GMF or NNMF conditions. 

In contrast, assessing the effects of GMF on the collective foraging behavior (Phase 2) shows that both the locomotory and the ability to perceive or release pheromone trails are GMF-dependent, as both the time and the number of misrouting events (errors) increase in NNMF (see also Section 2.2 for further discussion). 

### 2.2. The GMF Modulates Lasius niger Brain Biogenic Amines

**Figure 4:**
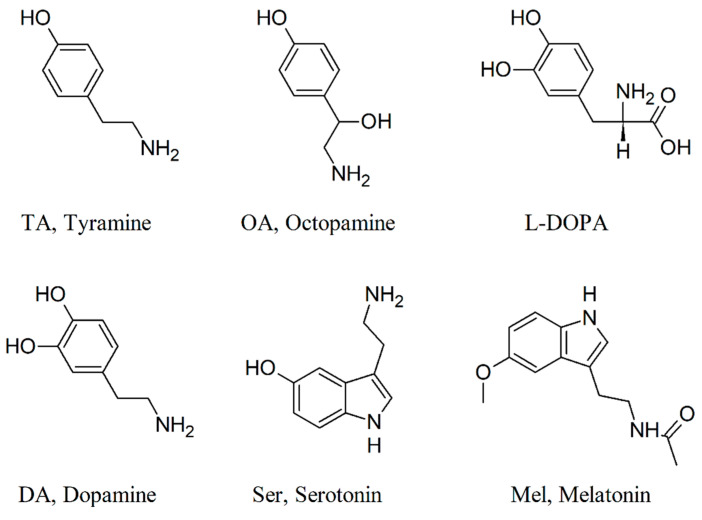
Chemical structure of the BAs analyzed in *L. niger* brain.

Biogenic amines (BAs) are compounds derived from amino-acid decarboxylation [52]. Although many of these compounds are known to function as toxins, some BAs are physiologically produced by organisms and function as key factors in several biological processes [42,53]. BAs are extremely diverse, but only a few have shown physiological importance in invertebrates, playing a different role with respect to vertebrates [35,37,38,54]. Consequently, we investigated whether the modulation of ant locomotor abilities by GMF, which affects the foraging performance, was correlated to qualitative or quantitative changes of the BA profiles in *L. niger* brains. We evaluated the content of those BAs that are known to modify invertebrate behavior, such as TA, OA, DA, L-DOPA, Ser, and Mel, using HPLC analysis coupled to mass spectrometry. Figure 4 shows the chemical structure of the BAs under study.

**Figure 5:**
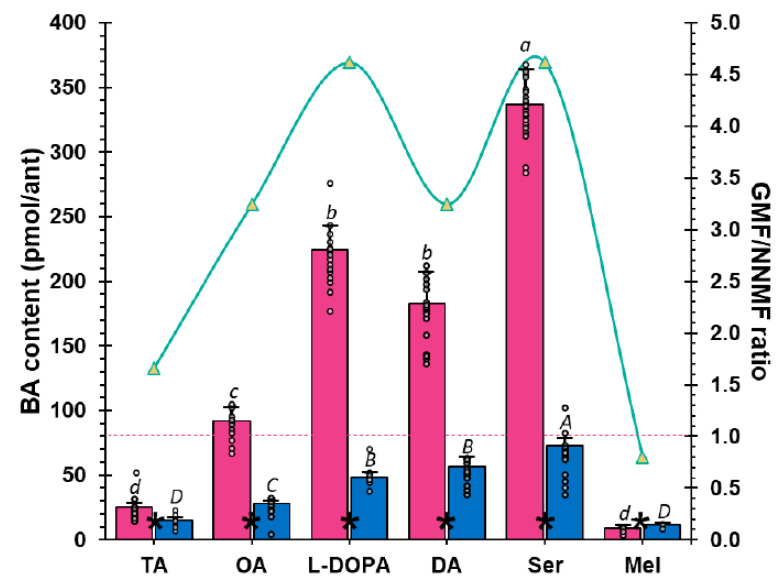
Differences in the average content of tyramine (TA), octopamine (OA), L-DOPA, dopamine (DA), serotonin (Ser), and melatonin (Mel) in black garden ants (*Lasius niger*) upon exposure to GMF (magenta bars) and NNMF (blue bars). Mean values are expressed as pmol·ant brain^−1^ (N = 30). For each series (GMF and NNMF), different letters indicate significant differences (*p* ≤ 0.05), as measured by Tukey's multiple interval test. The interpolation red line displays the GMF/NNMF ratio. The asterisk indicates a statistical difference between GMF and NNMF in the content of the relative BA. Metric bars indicate standard deviation. For further statistical analysis, see Supplementary Table S2.

Exposure to NNMF did not produce any qualitative difference in the BA profile of *L. niger* brains with respect to GMF (Figure 5). In both experimental conditions, Ser was the most produced BA. From a quantitative point of view, a significant (*p* < 0.05) reduction in TA, OA, L-DOPA, DA, and Ser was found in the brain of *L. niger* ants exposed to NNMF. On the other hand, the content of Mel was significantly (*p* < 0.05) lower in GMF-exposed ants (Figure 5). Specifically, most BAs were 3.2- to 4.6-fold higher under GMF than NNMF conditions (Figure 5). These findings suggest a significant role of the GMF in the production of almost all BAs. Although a direct causal effect has rarely been demonstrated, the correlation between the variation of BA levels and behavioral plasticity has been shown in various animal species [34]. These correlations are species- and context-dependent, making it difficult to assess a unique function for each BA. However, it is assumed that TA and OA may be primarily involved in regulating muscle contraction, sense organ sensitivity, and nestmate recognition [37,38,47]; DA and L-DOPA may be associated with aggressive behaviors and locomotor activity, while Ser is supposedly involved in ants’ chemical trace responsiveness [55]. A few studies described the role of Mel in invertebrates, and its presence has been confirmed only recently in insects, linked to abiotic stress tolerance [39,42]. The significant decrease in TA and DA contents observed in NNMF conditions (Figure 5) might be associated with a locomotor inhibition, as observed in several insects, including ants [38], and might explain why NNMF-exposed ants experienced altered foraging performances, as shown in our bioassays (Figure 1 and Figure 2). Indeed, the behavior of *L. niger* exposed to NNMF is a general decrease in locomotor capacity and lower effectiveness in perceiving chemical cues. The significant decrease found in the content of Ser and OA in ants exposed to NNMF with respect to GMF might be responsible for the reduction in chemical perception because both compounds are primarily involved in sensing, while Ser is known to specifically modulate ant responsiveness to pheromone trails [47,55,56]. Overall, these findings suggested that GMF is necessary for *L. niger* orientation by modulating the ant brain BA contents.

### 2.3. MagR Is a Highly Conserved Protein in Ants

MagR is an evolutionarily ancient protein, identified for the first time in *Drosophila* CG8198, and consists of an iron–sulfur complex (ISCA). ISCA proteins are involved in a model of light–magnetism-coupled magnetoreception that shows both the magnetic properties of Fe–S proteins and the light-dependent properties of cryptochrome (Cry) [19]. MagR has recently been designated as a potential magnetoreceptor protein and consists of a linear polymerization of Fe–S clusters forming a rod-shaped biocompass surrounded by the photoreceptor Cry [43]. Cry acts as a photoreceptor to receive light signals, while MagR acts as a magnetic receptor within the complex to detect magnetic signals and complete the sensing of geomagnetic information through the opto-magnetic coupling mechanism [4]. The MagR/Cry complex is consequently referred to as the magnetosensory complex (MagS). Recently, the protein has been shown to participate in several biological processes in plants and animals, including iron delivery, sensory redox, electron transport in respiration, photosynthesis, nitrogen fixation, and DNA replication or repair [22].

**Figure 6:**
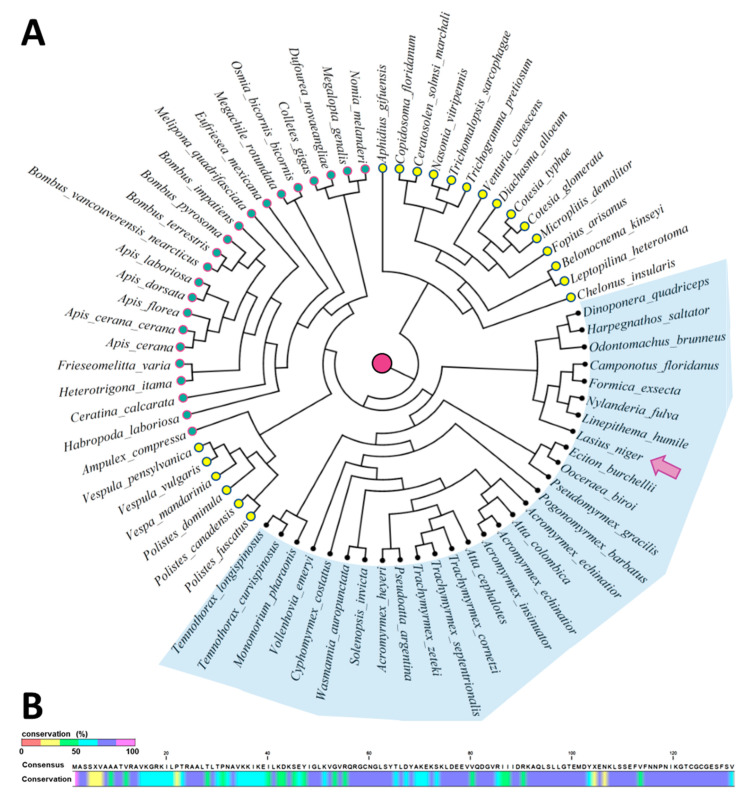
Phylogenetic analysis of the amino-acid sequence of MagR protein. (**A**) Phylogenetic tree of MagR originated from differences in amino-acid composition between *Drosophila melanogaster* (magenta dot in the center) and proteins identified in ants (blue shadow), bees (green dots), and wasps (yellow dots). The circular tree shows that the protein amino-acid sequences of phylogenetically close organisms are highly conserved, as suggested by their linking distance. The circular tree was originated by downloading the amino-acid sequences from NCBI, while the distance of each entry was calculated using BLAST tool as described in Section 3. (**B**) Sequence alignment of MagR in ants, wasps, and bees. Highly conserved residues among the analyzed species are shown in dark blue (>75% and <90%) or pink (>90%), while lowly conserved motif residues are colored in red, yellow, or green according to the conservation percentage (see percentage bar color legend).

To evaluate whether *MagR* could be expressed in ants and to identify conserved motifs in the amino-acid sequence, a phylogenetic analysis of homologous proteins was conducted, considering MagR from *Drosophila melanogaster* as the original entry. In addition, to better appreciate potential differences in sequence composition, the investigation also included analysis of species phylogenetically close to ants, namely, wasps and bees. By analyzing the sequences of MagR homologs of different ant, wasp, and bee species (Supplementary Data S1), we obtained a circular cladogram phylogenetic tree based on the inclusion of 96 amino-acid residues and exclusion of ambiguous positions (pairwise deletion option). Interestingly, the MagR protein was detected in both ants, wasps, and bees (Figure 6A). The amino acid composition resulting from an overall alignment of the sequences reported in Supplementary Data S1 is also reported (Figure 6B). Several similarities were found among bees, wasps, and ants. However, although many portions of the *MagR* gene were found to be highly conserved between species, some differences in amino-acid composition allowed the separation of ants from the other organisms (Figure 6A, blue shaded area). Moreover, although not as marked as for ants, wasps and bees were also found to be distinctly divided into different clusters for most of the cases (Figure 6A). Regarding the conserved regions, the amino-acid sequences of MagR of the species under study showed highly conserved sequence residues. In particular, four aromatic residues able to carry electrons (Y64, F112, F114, and F130) were 88% conserved during the evolution of MagR (Figure 6B). This result is in agreement with recent studies on the amino-acid sequence similarity of MagR in different organisms, suggesting that the position of these residues, their spacing between each other, and their location in the three-dimensional structure of the protein are functional for physiological electron tunneling ranging from 6 Å to 14 Å between the two neighboring residues [57]. This also indicates a possible role in intermolecular electron transport within the discoidal tetramer of MagR based on Marcus’s theory of electron transfer [57]. Moreover, in agreement with [57], we found two other residues (Y69 and Y104) to be highly conserved (Figure 6B). Notably, Y69 is tightly localized at the interface between Cry and the MagR tetramer, hypothesizing that it may contribute to electron transfer from/to Cry [57].

### 2.4. GMF Influences the Expression of MagR and Cry in Lasius niger

In order to investigate whether the GMF could affect the modulation of *MagR* and *Cry*, gene expression analysis was conducted in *L. niger* by qRT-PCR. In addition, because it was recently supposed that the effects derived from changes in MF might be a consequence of altered redox state ratio of *Cry* during photocycle [20,44,57], the expression of genes coding for enzymes responsible for cellular redox balance (*cSOD, mSOD, eSOD, eSOD2, CAT, GPX,* and *GSR*) was also assayed. Expression analysis on target genes was performed by first assessing the stability of reference genes under GMF or NNMF conditions. Specifically, the expression of *β-actin*, *ef-1β*, and three different *GADPH* isoforms was monitored. All the analyzed candidate reference genes were quite stable under GMF or NNMF conditions. Among them, *ef-1β* had a variation percentage of over 10% (Supplementary Figure S1). Our data point out that *β-actin* is the most suitable for the study of gene expression variation in *L. niger* ants under NNMF conditions.

**Figure 7:**
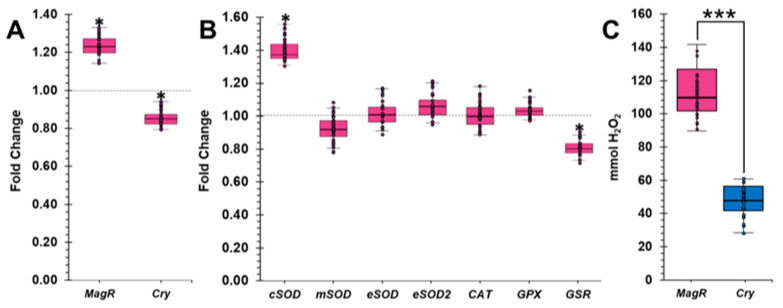
Effect of GMF on the gene expression of *MagR* and *Cry* (**A**), SOD, CAT, GPx, and GSR (**B**), or H_2_O_2_ content (**C**) of *Lasius niger*. For Panels A and B, Gene expression is expressed as fold change calculated as the GMF/NNMF ratio (N = 30). The dotted line represents the gene expression under NNMF condition, while bars represent the mean ± SD of three qRT-PCR biological replicates carried out in triplicate. For all panels, within each box the horizontal black lines indicate median values, while boxes extend from the 25th to the 75th percentile of the distribution of values in each group (N = 30). The extended vertical lines indicate the standard deviations. Asterisks indicate significant (* *p* < 0.05; *** *p* < 0.001) differences.

Regarding *MagR* and *Cry* expression, exposure to GMF increased the transcriptional levels of *MagR* 1.25-fold, while the expression of *Cry* was reduced 0.86-fold, with respect to NNMF (Figure 7A). 

Our analyses suggest that, although MagR is the only portion of MagS capable of perceiving MF intensity and orientation [19], at the transcriptional level, *Cry* appears to be oppositely influenced by variations in the MF, with respect to *MagR*. However, the MF may somehow influence the gene regulation and/or enzymatic activity of proteins deputed to cellular redox balance [58]. Because Cry is a protein susceptible to altered redox state, it is very likely that different regulation of *Cry* under GMF or NNMF conditions is a consequence of disturbances of reactive oxygen species (ROS) production.

### 2.5. GMF Modulates the Oxidative Stress of Lasius niger

In order to investigate whether the GMF could influence the expression of genes coding for enzymes involved in the cellular oxidative balance, the expression of genes coding for isoforms of superoxide dismutase (SOD) (*cSOD*, *mSOD*, *eSOD*, and *eSOD2*), catalase (*CAT*), glutathione peroxidase (*GPX*), and glutathione reductase (*GSR*) were assayed, under both GMF and NNMF conditions. SOD catalyzes the dismutation of the superoxide radical (O_2_^−^) into molecular oxygen (O_2_) and hydrogen peroxide (H_2_O_2_). Several isoforms of SOD are constitutively present in almost all living organisms. Although functionally similar, the isoforms have different cellular localization, and the study of their differential expression can provide useful insights into the location of oxidative stress [59]. GMF induced a significant (*p* < 0.05) upregulation of soluble cytoplasmic *SOD* (*cSOD*) isoform, whereas no significant difference was found for the other isoforms of SOD (Figure 7B). H_2_O_2_ detoxification is catalyzed by CAT and GPX [60]. Unlike CAT, GPX needs to be reduced by GSR to exert its function [61]. Gene expression analyses showed that *CAT* and *GPX* expression was unaffected by MF variations. In contrast, *GSR* was downregulated by the GMF (Figure 7B). These data suggest a potential upregulation of genes coding for enzymes that increase the H_2_O_2_ content in the cytoplasmic compartment.

In order to validate this hypothesis, we quantified the H_2_O_2_ content of ants exposed to either GMF or NNMF. Our data showed that *L. niger* ants under GMF conditions produced a higher amount of H_2_O_2_ with respect to NNMF, with a 45% reduction (Figure 7C). In addition to the effect of GMF on genes expressing for enzymes involved in ROS production, the reduced H_2_O_2_ content under NNMF condition might be associated with the increased amount of Mel (see Figure 5). Indeed, among the detected BAs, Mel is the only one exerting a strong antioxidant power, and, in vertebrates, it counteracts cellular oxidative stress [39,42]. The high antioxidant power of Mel has been attributed not only to its radical-scavenging activity but also to its degradation products [42]. Metabolism and/or catabolism mechanisms of Mel in invertebrates, including ants, are not fully disentangled; however, studies are underway and will be reported soon.

## 3. Materials and Methods

### 3.1. Animal Material and Experimental Conditions

Nests of *Lasius niger* were collected in June 2021 near the Stura river at Borgaro Torinese (45°09′29.7″ N, 7°38′15.6″ E; MF data: declination: 2.56’ E, inclination: 60.95’ down, intensity: ~42 µT). After collection, the nests were brought to the laboratory, where the species identification was confirmed using the key according to [62]. In the laboratory, the colonies were placed in plastic containers (45 × 35 × 30 cm; L × W × H) with the soil removed during collection. Colonies were fed three times a week, offering them a 10% (w/v) solution of honey, dead *Galleria mellonella* larvae, and water ad libitum. Before starting the training period (see Section 3.2.2), two groups (hereafter subsets), each containing 75 workers from each of the four colonies collected in the field were created. The workers of each subset were placed inside smaller plastic boxes (13 × 8 × 6 cm; W × L × H). A wet sponge, partially covered by a plastic lid, was added to each box to provide humidity and shelter. The colonies were supplied with water ad libitum. The lid of each box was perforated in the center to allow subsequent connection to the experimental foraging arena (see Section 3.2.1).

### 3.2. Locomotor Activity and Orientation Test

#### 3.2.1. Experimental Setup

The foraging arena was built to observe the foraging behavior of *L. niger*. The general structure of the experimental labyrinth includes two mirrored arms that begin from a common central square, representing the entrance to the arena. Each arm, in turn, divides itself into two other paths, which finally end with a T-shaped bifurcation (Supplementary Figure S2).

In detail, to build the arena, a 28 × 12 × 1cm transparent plexiglass rectangle was used as a support base for the experimental labyrinth, and a second, same-size rectangle was positioned above the latter to create a completely isolated artificial environment. A small hole was opened in the center of the plexiglass base and was used to connect the arena with the underneath nest through a wooden stick, allowing the workers to enter the labyrinth. The walls that compose the path of the arena were designed using the AutoCAD software (version 2022), and subsequently replicated with a 3D printer.

The food source, a solution of water and honey (10%), was always placed in the same position and aligned in the direction of the Earth's geomagnetic north. Therefore, hypothetically the direction chosen by the first workers entering the arena implies discrimination between north and south. The subsequent branches, on the other hand, allow workers to choose between different directional combinations, both alternating and repetitive (left–right: L/R/L; L/R/R; R/L/L; R/L/R).

#### 3.2.2. Training Procedure

Before behavioral observations, colonies were trained for 2 weeks to search for the food source within the arena. The training phase began 4 days after removing the food from the nest boxes, and, from this moment on, the food was provided exclusively in the manner described hereafter. The food source was located inside the labyrinth in the same branch of the arena (used for the experimental phases, see Section 3.2.1) in such a way as to allow the workers to memorize the series of correct directional choices, which led from the center of the arena to the source of food. The food source was located in the direction of the Earth's geomagnetic north. Assuming that the ants can orient themselves according to the geomagnetic field, the workers should have associated the food source with a specific position relative to the magnetic compass. A blue landmark was placed in the upper part of the plexiglass roof of the arena, near the food source, to also offer the foragers a visual signal that could be used as a spatial reference. The color was chosen according to literature data showing that blue and yellow are particularly distinguishable by ants [63].

#### 3.2.3. Behavioral Tests

After the training phase, we conducted behavioral observations to verify the effect of the MF on the foraging activity of *L. niger*. We used the Triaxial Helmoholtz coil system to reduce the GMF to NNMF values as previously described [33]. The GMF conditions in the laboratory were as follows: declination, 2.56’ E; inclination, 60.95’ down; intensity, 42.20 ± 0.02 µT (see Supplementary Figure S3 for details). NNMF values ranged from 40 to 50 nT, with the same inclination and declination as the GMF. A three-axis sensor (model Mag-03, Bartington Instruments, Oxford, UK) was positioned at the center of the Helmholtz coils, in order to obtain a continuous real-time measurement of the intensity of the MF inside the chamber of exposure in which the experiments were conducted. 

One of the two subset colonies was tested in NNMF, while the other subset served as a control and was observed under GMF. To minimize the differences in other environmental variables, the observations of the controls were carried out within the triaxial Helmoholtz coil system, keeping the three Helmoholtz coils switched off. Each subset colony was observed seven times.

Before starting the observations, the sub-colonies were left inside the device for 10 min acclimatation, to avoid any type of influence related to preparation of the experimental setup.

The experimental protocol included two successive phases (Phase 1 and 2). 

*Phase 1.* The arena was connected to the artificial nest underneath using a wooden stick passing through the holes in the center of the plexiglass base and in the lid of the box constituting the nest. The stick had to be small enough to allow the workers to pass through the holes and reach the center of the arena. After one worker entered the arena, the stick was raised and positioned in such a way as to block the entrance to other workers. The first part of the observation ended when the scouting worker (referred to as the first worker) managed to find and use the food, and then returned to the center of the arena. Then, the nest entrance was reopened, to allow the first forager to leave the arena and transport the food within the nest. Phase 1 began when the first worker entered the arena (labyrinth) and ended when the forager returned to the nest after having found and fed on the food source.

*Phase 2.* After the first forager had returned to the nest, we allowed all the workers to enter the arena by positioning the stick in the opening position. Phase 2 ended after a fixed time of 15 min. This time limit was decided on the basis of observations made during the training, in which 15 min was more than sufficient to allow the workers to complete their foraging activity after the first worker had returned.

Video recordings of the experiments were performed with an E-M10 Mark Ⅳ digital camera (Olympus). The camera was positioned above the triaxial Helmoholtz coil system on a plexiglass panel. The camera was connected to a Hersmay PS-BLS5/BLS external power supply and used remotely via the Olympus Image Share App Version 4.5.1. For each subset, both phases were recorded in succession. At the end of each recording, the nest and the arena were removed from the triaxial Helmoholtz coil system, and the arena was washed with ethanol and then with water in order to remove the chemical traces released by the ants during the foraging activities. Immediately after the behavioral tests, 15 workers per three subsets in GMF and three subsets in NNMF (a total of 90 samples) were frozen in liquid nitrogen and stored at −80 °C for subsequent chemical (see Section 3.3) and genetic analysis (Section 3.5 and Section 3.6).

#### 3.2.4. Parameter Monitoring

After conducting and recording all the experimental tests, videos were processed using the software Boris v. 7.10.7 [64].

In Phase 1 and 2, six parameters were taken into consideration as reported in Supplementary Table S3. Time parameters were expressed as seconds, while errors made by workers when foraging or returning to the nest and contacts between workers as number of occurrences.

### 3.3. Extraction of Brain Biogenic Amines

BAs were extracted from *L. niger* as previously described [38]. Briefly, after a rapid beheading under a dissection microscope using micro-scissors, brains were extracted using 20 µL of 0.1% (*v*/*v*) HCl and 5 mM heptafluorobutanoic acid (HFBA). HFBA was used as an ion-pairing agent for the highly polar BAs. The extraction performance was monitored by adding a known amount of 2-phenylethylamine (PEA) to the solvent as internal standard. After homogenizing the ant brain with a micropaste within the extraction solvent, the solution was sonic-bathed for 10 min, and samples were centrifuged at 12,000× *g* for 20 min at 4 °C to separate solid from liquid components. Lastly, a solution of isopropanol/chloroform (1:4, *v*/*v*) was directly added to the supernatant in a 1:1 (*v*/*v*) ratio to remove undesirable compounds. Samples were centrifuged at 12,000× *g* for 10 min at 4 °C, and the aqueous upper-phase obtained from centrifugation was immediately injected into an HPLC–ESI-MS/MS (1200 HPLC, Agilent Technologies, Santa Clara, CA, USA) for quantification of BAs. Elution of BAs was performed at 0.2 mL·min^−1^ by a solvent gradient composed of 5 mM HFBA in H_2_O (Solvent A) and 5 mM HFBA in MeOH (Solvent B) on a Luna C18(2) reversed-phase column (150 mm × 3 mm, particle size 3 µm, pore size 100Å, Phenomenex, Bologna, Italy). The gradient was kept in isocratic conditions (20% of Solvent B) for the first 2 min; then, it reached 50% Solvent B in 1 min. From 3 to 10 min, the gradient was readjusted reaching 100% Solvent B and maintained for 5 min. Finally, the chromatographic condition was reconditioned for 6 min using the starting condition. Regarding the mass spectrometer, source parameters were set as follows: nebulizing gas flow, 3 L·min^−1^; desolvation line temperature, 250 °C; heat block temperature, 400 °C; drying gas flow, 10 L·min^−1^. The mass spectrometer operated in MRM positive ion mode, monitoring the transitions of 138.0 > 121.0 (tyramine; RT: 14.3 min), 154.0 > 137.0 (dopamine; RT: 16.8), 154.0 > 137 (octopamine; 15.3), 177.0 > 160.0 (serotonin; RT: 21.1), 198.0 > 181.0, 152.0 (L-DOPA; RT: 18.8), and 233.0 > 216.0, 191.0, 174.0 (melatonin; RT: 18.9). All standards used were purchased from MERCK (Germany).

### 3.4. Protein Phylogenetic Analysis

To find homologous sequences of the MagR protein across ants and closely related organisms (wasps and bees), a phylogenetic analysis was conducted as previously described [65], using the *Drosophila melanogaster* MagR amino-acid sequence as the original entry (Accession number: NP_573062.1). The database containing the sequences was completed in December 2022, and the search was conducted online at the National Center for Biotechnology Information (NCBI; https://www.nih.gov/, accessed on 5 December 2022) website. A BLASTp search was performed to search for MagR in ants, wasps, and bees, using nonredundant protein sequences (nr) as queries and limiting the query to organisms contained in specific libraries (taxid: 7399 and 7400). The search algorithm was then adjusted by setting the threshold for the expected number of random matches in a random pattern (expected threshold) to 0.05 and the seed length that initiates an alignment (word size) to 6. Regarding the scoring parameters, the BLOSUM62 matrix was used, and the gap cost for creating and extending a gap in an alignment was set to 11 and 1, respectively. After obtaining the database, all nonredundant results of these searches with E values ≤1 × 10^−5^ were extracted through an iterative screening process. The putative amino-acid sequences of the selected organisms were then used to perform a phylogenetic analysis using NCBI TreeViewer (ver. 1.19.4) to compute the distance tree of the results [65]. Hierarchical protein classification was performed by the neighbor joining method, with a maximum difference of 0.85, and arranged according to Grishin's visualization.

### 3.5. Gene Expression Analysis by qRT-PCR 

In order to perform gene expression analysis on ant samples, RNA was extracted and reverse-transcribed. Briefly, *L. niger* was rapidly beheaded under a dissection microscope using micro-scissors, and, in order to prevent contamination by retinal pigments, a small medial–lateral incision was made directly behind the mandibles to remove the optic lobes. Brain RNA was extracted using an RNeasy Mini Kit R (Qiagen, Hilden, Germany). The quality and quantity of total RNA were checked using both a UV/visible nano-spectrophotometer (BioSpecnano, Shimadzu, Japan) and 1% (*w/v*) agarose gel electrophoresis. The isolated RNA was used as template for reverse transcription (cDNA Maxima H Minus First Strand, Thermo Fisher Scientific, Waltham, MA, USA), following the manufacturer’s instructions. The obtained cDNA was consequently used for quantitative real-time PCR by a QuantStudio 3 (Thermo Fisher Scientific, Waltham, MA, USA), Maxima SYBR Green qPCR Master Mix (Thermo Fisher Scientific, Waltham, MA, USA), and using the primers reported in Supplementary Table S4. After evaluating the stability of each reference gene (*GAPDH1, GAPDH2, GAPDH3,* and *β-Actin*) under the different experimental conditions, the expression levels of the target genes (*GPX, GSH, SOD1, SOD2, SOD3, SOD4*, *MagR*, and *Cry*) were calculated using the Pfaffl method [66]. Primers for target and reference genes were designed with Primer3 v.4.1.0 software.

### 3.6. Hydrogen Peroxide Quantification 

H_2_O_2_ content was measured using the MAK311 Peroxide Assay kit (Sigma-Aldrich, St. Louis, MI, USA) according to the manufacturer’s instructions. Briefly, whole ants exposed to GMF or NNMF were ground using a micropestle and extracted in milliQ water using a 1:2 (*w*/*v*) ratio. After centrifugation (15,000× *g*; 10 min; 4 °C), 4 uL of limpid supernatant was injected into 20 µL of reaction buffer provided by the manufacturer in the commercial kit. Simultaneously, 4 µL of hydrogen peroxide (Sigma-Aldrich, St. Louis, MI, USA) at different standard concentrations was incubated in 20 µL of the same reaction buffer in order to make a calibration curve (LOD: 1.2 mmol LOQ: 3.5 mmol; R^2^: 0.9998; y = 0.0246x + 0.0375). Both ant extracts and wells containing H_2_O_2_ at different concentrations were incubated for 30 min at room temperature. The absorbance resulting from the reaction was monitored at 585 nm using a BioSpec-nano spectrophotometer (Shimadzu, Kyoto, Japan).

### 3.7. Statistical Analysis 

Kolmogorov–Smirnov tests were used to assess data distribution. Results were expressed as the mean ± standard deviation (SD).

To verify if the presence/absence of the geomagnetic field affects the behavioral variables, we computed generalized linear mixed models with a negative binomial distribution to account for overdispersion including the colony identity as random factor using the glmer.nb function of the lme4 R package. In a few cases, where the assumptions were satisfied, we computed linear mixed models including the colony identity as random factor. For each model, the likelihood ratio chi-square was calculated using the function Anova in the car package.

To understand which factors could influence the time spent by the foragers to return to the nest during Phase 2, we performed model selection using the dredge function from the MuMIn package, starting with a full model that included the presence/absence of the geomagnetic field, the number of errors and their interaction, and the sequential order of arrival of each forager. The colony identity was included as a random factor. We selected equally plausible models with ΔAICc < 4. For the best model, the likelihood ratio chi-square was calculated.

The graphs related to the behavioral observations were produced using the ggplot2 R package.

ANOVA followed by Tukey’s post hoc test was applied to determine significant differences in biogenic amine and transcriptomic data using SPSS Statistics ver. 29 (SPSS, Chicago, IL, USA). 

## 4. Conclusions

Overall, the results obtained in our study showed that the GMF is necessary to enhance the efficiency of *L. niger* foraging by decreasing the time and mistakes made to discover the food source and allowing a quicker return to the nest. These results are consistent with other studies (see [46] and reference therein) that observed the disorientation of ants after exposure to altered MF (e.g., [47]). Interestingly, we observed a decrease in the orientation performance in NNMF, despite the presence of all other signals (chemicals and visuals) used to find their bearings. Therefore, our findings suggest that the GMF is an essential orientation cue for *L. niger* even if landmarks or trail compounds are available. 

Our BA analyses shed light on the potential mechanism through which the GMF might affect ant orientation. Indeed, in NNMF, the reduction in BAs that are known to be correlated with a decrease in the locomotor ability (TA and DA) and chemical perception of trails (OA and Ser) is associated with a longer time and higher misrouting events occurring while *L. niger* searches for food or homing. 

Although the examination of the mechanism of MF perception in ants was beyond the scope of our work, our results confirm the modulation of *MagR* and *Cry* in *L. niger* and the regulation of genes coding for enzymes involved in maintaining the cellular oxidative state. In GMF-exposed ants, the upregulation of both *SOD* and the concomitant downregulation of *GSR* agreed with the increasing content of H_2_O_2_, suggesting increased oxidative stress in *L. niger* exposed to GMF throughout the time of the experiment. As recently demonstrated in *Arabidopsis thaliana* [44], these results indicate that the GMF induces a basic oxidative stress, characterized by the modulation of genes that contribute to ROS production. This circumstance has been hypothesized to generate a mild oxidative stress condition that organisms evolved in a GMF environment. Changes in MF would then induce changes in the redox status in *L. niger*, indicating a functional role of ant magnetoperception in response to stress. 

The GMF was present long before any organism evolved on Earth. Our results provide new insights into an emerging field of research trying to understand how the GMF contributed to life evolution and how invertebrates used GMF variations to infer directions and positions. Here, we showed that *L. niger* is able to orient itself in a GMF-dependent manner, and we demonstrated that this ant species possesses the magnetosensory complex to perceive MF variations. We hypothesize that GMF modulates ant behavior by interfering with the aminergic activity of their brains.

## Data Availability

Data are available upon request to the corresponding author.

## Supplementary Materials

The following supporting information can be downloaded at https://www.mdpi.com/article/10.3390/ijms24054387/s1.
