# Supplementary material for: The Geomagnetic Field (GMF) Is Necessary for Black Garden Ant (Lasius niger L.) Foraging and Modulates Orientation Potentially through Aminergic Regulation and MagR Expression
Source: Int J Mol Sci. 2023 Feb 23;24(5):4387. doi: 10.3390/ijms24054387 (PMC10002094; doi:10.3390/ijms24054387)
Supplement: Supplementary file 1 [file ijms-24-04387-s001.zip › Supplementary Figure S1.docx]

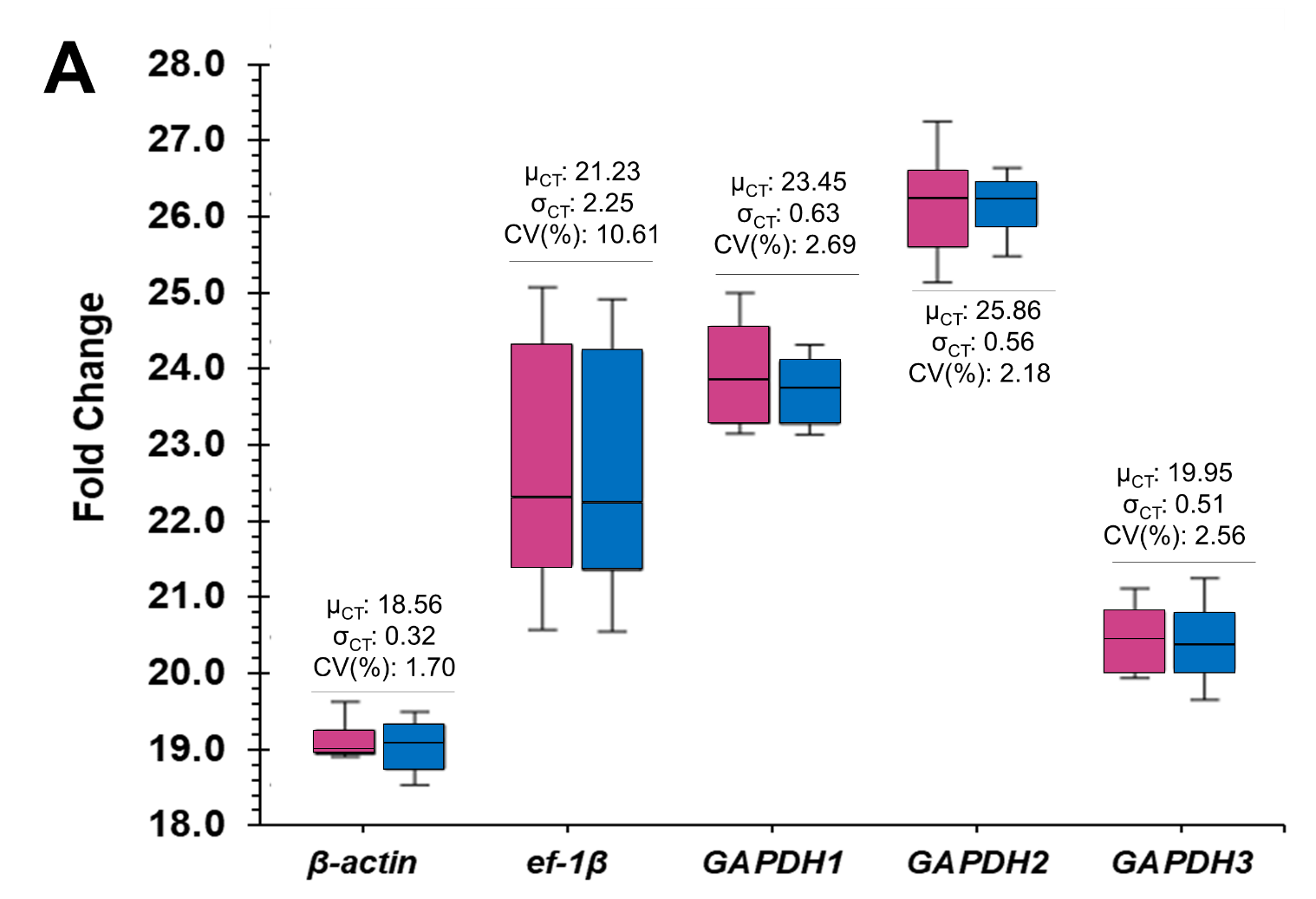


**Supplementary Figure 1**: **Range of cycle threshold (Ct) values of the five candidate reference gene (*β-actin, ef-1β, GAPDH1, GAPDH2, and GAPDH3*) in *Lasius niger* ants under GMF (magenta box) or NNMF (blue box) condition.** Within each box, horizontal black lines indicate median values, while boxes extend from the 25th to the 75th percentile of the distribution of values in each group. The extended vertical lines indicate the standard deviations. For each housekeeping gene, mean value (μ), standard deviation (σ), and variation coefficient (CV%) is reported considering GMF and NNMF as a single variable.
