# Supplementary material for: The Geomagnetic Field (GMF) Is Necessary for Black Garden Ant (Lasius niger L.) Foraging and Modulates Orientation Potentially through Aminergic Regulation and MagR Expression
Source: Int J Mol Sci. 2023 Feb 23;24(5):4387. doi: 10.3390/ijms24054387 (PMC10002094; doi:10.3390/ijms24054387)
Supplement: Supplementary file 1 [file ijms-24-04387-s001.zip › Supplementary Figure S3.docx]

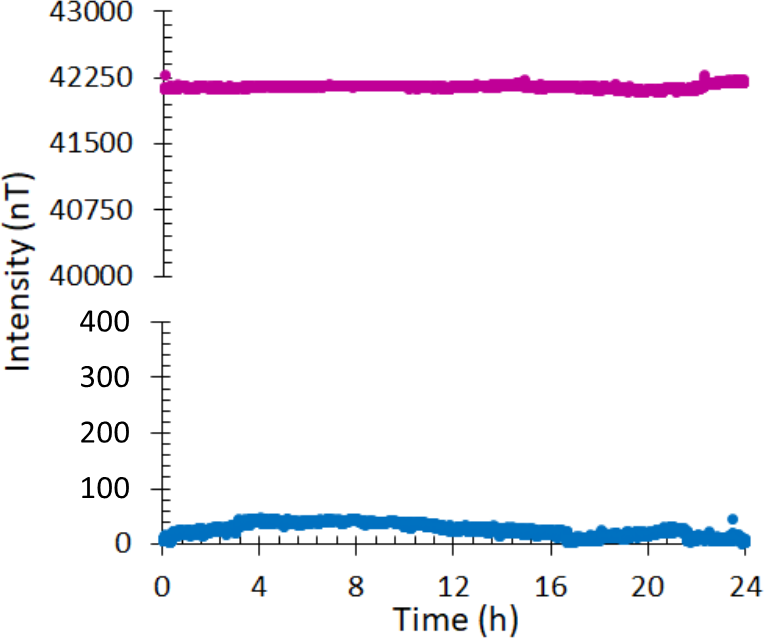


**Supplementary Figure 3: Time-course variations of the magnetic field intensity.** The intensity is expressed as nT inside the triaxial Helmholtz coils system with current (blue line) and without current (magenta line) in the coils. Data are representative of 24 h.
