# Supplementary material for: The Geomagnetic Field (GMF) Is Necessary for Black Garden Ant (Lasius niger L.) Foraging and Modulates Orientation Potentially through Aminergic Regulation and MagR Expression
Source: Int J Mol Sci. 2023 Feb 23;24(5):4387. doi: 10.3390/ijms24054387 (PMC10002094; doi:10.3390/ijms24054387)
Supplement: Supplementary file 1 [file ijms-24-04387-s001.zip › Supplementary Table S1.docx]

**Supplementary Table 1: Models.** Model selection using the dredge function (Barton, 2015) based on the Akaike information criterion corrected (AICc) ranking the influence of sequence rank, magnetic field, errors, and the combination of the last two factors on the time (s) to return to the nest during the second phase. Colony identity was included as the random variable. We only report models equal or lower than ΔAICc = 4. Degrees of freedom of the model (d.f.), differences in AICc-values (ΔAICc) and Akaike weight (ω) are shown.

|  | (int) | **magnetic field** | **worker order** | **errors** | **magnetic field x errors** | df | logLik | AICc | ΔAICc | ω |
| --- | --- | --- | --- | --- | --- | --- | --- | --- | --- | --- |
| 16 | 2.413 | + | -0.007716 | 0.2562 | + | 7 | -1732.312 | 3478.9 | 0 | 0.333 |
| 8 | 2.447 | + | -0.008525 | 0.2034 |  | 6 | -1733.395 | 3479 | 0.11 | 0.315 |
| 14 | 2.326 | + |  | 0.2672 | + | 6 | -1733.91 | 3480 | 1.14 | 0.188 |
| 6 | 2.355 | + |  | 0.2068 |  | 5 | -1735.376 | 3480.9 | 2.02 | 0.121 |
