# Supplementary material for: The Geomagnetic Field (GMF) Is Necessary for Black Garden Ant (Lasius niger L.) Foraging and Modulates Orientation Potentially through Aminergic Regulation and MagR Expression
Source: Int J Mol Sci. 2023 Feb 23;24(5):4387. doi: 10.3390/ijms24054387 (PMC10002094; doi:10.3390/ijms24054387)
Supplement: Supplementary file 1 [file ijms-24-04387-s001.zip › Supplementary Table S2.docx]

**Supplementary Table 2: Post hoc tests**. Hsd post hoc differences in biogenic amines (BAs) under GMF or NNMF condition.

|  | | **GMF** | **NNMF** |
| --- | --- | --- | --- |
| **TA** | *TA* | 13.1193* | 66.6129* |
|  | *L-Dopa* | 33.5015*** | 199.4396*** |
|  | *DA* | 41.2729*** | 158.0404*** |
|  | *SERO* | 57.8077*** | 311.691*** |
|  | *MELA* | 3.723 | 15.9397 |
| **OA** | *TA* | 13.1193* | 66.6129* |
|  | *L-Dopa* | 20.3821** | 132.8267*** |
|  | *DA* | 28.1536*** | 91.4275*** |
|  | *SERO* | 44.6883*** | 245.0781*** |
|  | *MELA* | 16.8423* | 82.5527** |
| **LDA** | *TA* | 33.5015*** | 199.4396*** |
|  | *L-Dopa* | 20.3821** | 132.8267*** |
|  | *DA* | 7.7714 | 41.3991 |
|  | *SERO* | 24.3061*** | 112.2514*** |
|  | *MELA* | 37.2245*** | 215.3794*** |
| **DA** | *TA* | 41.2729*** | 158.0404*** |
|  | *L-Dopa* | 28.1536*** | 91.4275*** |
|  | *DA* | 7.7714 | 41.3991 |
|  | *SERO* | 16.5347* | 153.6506*** |
|  | *MELA* | 44.996*** | 173.9802*** |
| **SERO** | *TA* | 57.8077*** | 311.691*** |
|  | *L-Dopa* | 44.6883*** | 245.0781*** |
|  | *DA* | 24.3061*** | 112.2514*** |
|  | *SERO* | 16.5347* | 153.6506*** |
|  | *MELA* | 61.5307*** | 327.6308*** |
| **MELA** | *TA* | 3.723 | 15.9397 |
|  | *L-Dopa* | 16.8423* | 82.5527** |
|  | *DA* | 37.2245*** | 215.3794*** |
|  | *SERO* | 44.996*** | 173.9802*** |
|  | *MELA* | 61.5307*** | 327.6308*** |
