# Supplementary material for: The Geomagnetic Field (GMF) Is Necessary for Black Garden Ant (Lasius niger L.) Foraging and Modulates Orientation Potentially through Aminergic Regulation and MagR Expression
Source: Int J Mol Sci. 2023 Feb 23;24(5):4387. doi: 10.3390/ijms24054387 (PMC10002094; doi:10.3390/ijms24054387)
Supplement: Supplementary file 1 [file ijms-24-04387-s001.zip › Supplementary Table S3.docx]

**Supplementary Table 3: The ethogram.** Variables collected in behavioral observation during foraging activities.

| **Foraging phase** | **Code** | **Behavior** |
| --- | --- | --- |
| Phase 1 | t1 | the time (s) spent by the first forager to enter the arena |
|  | t2 | the time (s) spent by the first forager to reach the food source |
|  | t3 | the time (s) spent by the first forager at the food source |
|  | t4 | the time (s) spent by the first forager to return to the nest |
|  | x1 | the number of mistakes made by the first forager to reach the food source |
|  | x2 | the number of mistakes made by the first forager to return to the nest |
| Phase 2 | t5 | the time (s) spent by the first recruited forager to reach the food source |
|  | wn | the time (s) spent by the workers to reach the center of the arena |
|  | wm | the number of errors made by the foragers to return to the center of the arena |
|  | si | the number of instantaneous interactions (less than three seconds) between two workers |
|  | sp2 | the number of long-lasting interactions between two workers |
|  | sp2time | the time (s) of long-lasting interactions between two workers |
