# Supplementary material for: The Geomagnetic Field (GMF) Is Necessary for Black Garden Ant (Lasius niger L.) Foraging and Modulates Orientation Potentially through Aminergic Regulation and MagR Expression
Source: Int J Mol Sci. 2023 Feb 23;24(5):4387. doi: 10.3390/ijms24054387 (PMC10002094; doi:10.3390/ijms24054387)
Supplement: Supplementary file 1 [file ijms-24-04387-s001.zip › Supplementary Table S4.docx]

**Supplementary Table 4**: **Primers**. PCR primer sequences used in quantitative real-time PCR analysis.

| **Protein** | | **Sequence (5'-3')** | |
| --- | --- | --- | --- |
| *CuZnSOD (or cytoplasmatic SOD)* | *SOD1* | **F** | GGCTTACACGGCTTTCATGT |
|  |  | **R** | ACCAGCTTCCACGTTTCCTA |
| *MnSOD (or mitocondrial SPD)* | *SOD2* | **F** | ACGTCAACACACAAATCGCT |
|  |  | **R** | TCATATCCAAGCCAACCCCA |
| *CuZnSOD (or extracellular SOD)* | *SOD3* | **F** | CCCACTTCAATCCCGACAAC |
|  |  | **R** | CACCAATAACACCACAGGCC |
| *SOD4 (or extracellular SOD)* | *SOD4* | **F** | GGGCTTCATGGTTTTCACGT |
|  |  | **R** | CCGGTCAGCGAGATCATACT |
| *Catalase* | *CAT* | **F** | GCATGCGAACTATCAGCCAA |
|  |  | **R** | GTACAAACGACCTGCCTGTG |
| *Glutathione peroxidase* | *GPX* | **F** | TGCCTGATCGTAAATGTCGC |
|  |  | **R** | CCGCCCTTCTCTTGTTTCAG |
| *Glutathione Reductase* | *GSH* | **F** | GTTCTGGAAATGGACGGCAA |
|  |  | **R** | TCGCCTTCTTCTCCTCCTTC |
| *Magnetic Receptor* | *MagR* | **F** | TGAGAGTTACAGTGGAGGGC |
|  |  | **R** | ACTGCATCCCTGTTCAGCTA |
| *Cryptochrome* | *Cry* | **F** | CTACCAGCTCACCGATCTGT |
|  |  | **R** | GTCTGACCGTTTGCCCATTT |
| β-actin | *β-actin* | **F** | CTCTTCCAACCCTCGTTCCT |
|  |  | **R** | GCGGGGCGATAATCTTGATC |
| *Elongation Factor 1β* | *ef-1β* | **F** | AGCGAAGATGACGATGACCT |
|  |  | **R** | TTGTGGCTGTTGAGATGCAC |
| *Glyceraldehyde-3-Phosphate Dehydrogenase 1* | *GAPDH1* | **F** | CCCTCTGAACTTGCTCTGGA |
|  |  | **R** | TGACCACACTGACCTCTTCC |
| *Glyceraldehyde-3-Phosphate Dehydrogenase 2* | *GAPDH2* | **F** | TCGAAAACCCTACCGAACCA |
|  |  | **R** | ACTGCTTCCTGAACCTCCTT |
| *Glyceraldehyde-3-Phosphate Dehydrogenase 3* | *GAPDH3* | **F** | GTCGTCGCCATCAATGATCC |
|  |  | **R** | GCCTTCCAAGTGAGCAGATG |
